# Order Lot Sizing: Insights from Lattice Gas-Type Model

**DOI:** 10.3390/e27080774

**Published:** 2025-07-23

**Authors:** Margarita Miguelina Mieras, Tania Daiana Tobares, Fabricio Orlando Sanchez-Varretti, Antonio José Ramirez-Pastor

**Affiliations:** 1 San Rafael Regional Faculty, Institute of Applied Physics (INFAP), CONICET, National Technological University (UTN), Gral. Urquiza 314, San Rafael, Mendoza 5600, Argentina; mierasmiguelina@hotmail.com (M.M.M.); tanitobares@gmail.com (T.D.T.); 2 Department of Physics, Institute of Applied Physics, CONICET, National University of San Luis, Ejército de Los Andes 950, San Luis 5700, Argentina

**Keywords:** optimization, order lot-sizing problem, lattice gas model, exhaustive enumeration of states, configurational entropy

## Abstract

In this study, we introduce a novel interdisciplinary framework that applies concepts from statistical physics, specifically lattice-gas models, to the classical order lot-sizing problem in supply chain management. Traditional methods often rely on heuristic or deterministic approaches, which may fail to capture the inherently probabilistic and dynamic nature of decision-making across multiple periods. Drawing on structural parallels between inventory decisions and adsorption phenomena in physical systems, we constructed a mapping that represented order placements as particles on a lattice, governed by an energy function analogous to thermodynamic potentials. This formulation allowed us to employ analytical tools from statistical mechanics to identify optimal ordering strategies via the minimization of a free energy functional. Our approach not only sheds new light on the structural characteristics of optimal planning but also introduces the concept of configurational entropy as a measure of decision variability and robustness. Numerical simulations and analytical approximations demonstrate the efficacy of the lattice gas model in capturing key features of the problem and suggest promising avenues for extending the framework to more complex settings, including multi-item systems and time-varying demand. This work represents a significant step toward bridging physical sciences with supply chain optimization, offering a robust theoretical foundation for both future research and practical applications.

## 1. Introduction

The classical lot-sizing problem is a fundamental challenge in production and inventory management, where the goal is to determine optimal ordering quantities and timing to minimize total costs, including setup, holding, and shortage costs, over a planning horizon. Traditionally, this problem has been approached using deterministic and stochastic optimization techniques, such as dynamic programming and integer programming, which focus primarily on cost efficiency within a discrete planning framework [1,2,3,4,5,6,7,8,9,10,11,12,13,14,15].

Despite these advances, the application of thermodynamics and statistical physics concepts to the lot-sizing problem remains largely unexplored. The objective this study was to explicitly leverage statistical physical modeling techniques to develop a novel framework for analyzing and solving the lot-sizing problem. Specifically, we mapped the discrete ordering process onto a lattice gas model, an established construct in physics, allowing us to apply thermodynamic principles to analyze and optimize ordering strategies.

The methodology involves formulating the lot-sizing problem as an equivalent model of particles adsorbed on a lattice, where each potential order corresponds to an occupied site. Ordering decisions are treated as thermodynamic states, establishing a direct analogy with lattice gas models. A scenario with *N* orders over a planning horizon of *M* periods, incurring a fixed ordering cost $C_{O}$ per order and a holding cost $C_{H}$ per period, is represented as a lattice of *M* sites with *N* monomers, governed by a chemical potential μ. Within this representation, cost components such as setup and holding costs are expressed as energy terms in a Hamiltonian, enabling the use of statistical mechanics tools, such as cluster approximation and free-energy minimization, for analysis. Moreover, this formulation introduces configurational entropy as a meaningful metric to assess the complexity and robustness of inventory strategies, offering an additional dimension to guide optimization.

As mentioned in the previous paragraph, the methodology employed in this work involves the use of the cluster approximation (CA). CA is a brute-force method for estimating a system’s partition function based on the exact enumeration of microstates within small clusters [16,17,18,19,20,21]. Over the past three decades, our research group has extensively applied this technique to lattice gas models, developing systematic state enumeration strategies for systems involving spatial correlations and constrained interactions [22,23,24]. The present work extends these tools to the domain of supply planning and order scheduling, opening a new line of theoretical inquiry with potential applications well beyond the scope of traditional production models.

CA has proven to be effective across various physical systems, including adsorption [16,17,18,19], percolation [20], and magnetism [21]. Here, we demonstrate its utility in supply chain modeling, where it provides accurate predictions of critical quantities such as total cost and order distributions, closely aligning with known exact solutions [25].

Our main results reveal several key insights. First, optimal supply policies emerge naturally from the minimization of a free energy function, closely mirroring the principles governing equilibrium in physical systems. This establishes a foundational link between statistical physics and supply chain optimization. Second, the cluster approximation enables highly accurate estimations of cost-related metrics, validating the model against benchmark results. Third, configurational entropy is shown to serve as a powerful indicator of system complexity and robustness. In the specific case examined in [25], which involved a one-dimensional mechanical system of particles connected by elastic springs, the optimal cost-minimizing configuration, where all spring lengths were equal and net forces vanished, corresponded to a minimum in the configurational entropy, thereby validating the physical model through thermodynamic consistency. Lastly, our results show that statistical physics provides a compact and effective toolkit for analyzing discrete planning problems, with the discrete nature of the planning horizon *M* making the cluster approximation particularly well-suited for revealing the structure of optimal strategies.

In summary, this study bridges the domains of supply chain optimization and statistical physics, offering a rigorous, conceptually rich framework for developing flexible and robust ordering policies. By reinterpreting classical inventory models through the lens of thermodynamics, we introduce powerful analytical tools with the potential to transform how supply planning problems are approached and solved.

## 2. Literature Review

The optimal lot-sizing problem has been addressed through various classical approaches primarily focused on jointly evaluating ordering and holding costs as key decision criteria. One of the traditional models in inventory management is the economic order quantity (EOQ) model. This model was initially developed by Ford W. Harris in 1913 [26] and later refined by R.H. Wilson in 1934 [27]. Its core principle states that the optimal lot size is reached when total inventory holding costs and ordering costs are balanced. An extension of the EOQ is the Part-Period Balancing Model, which allows for replenishments at discrete time intervals. In this case, instead of using a fixed lot size, the order quantity for each period is calculated based on the remaining inventory level and the projected demand for the following period [28]. Among classical models, the Wagner–Whitin algorithm also stands out [29]. This dynamic programming approach aims to minimize total costs over a finite planning horizon by considering both ordering and holding costs, without assuming constant lot sizes.

There are also heuristic methods to address this problem, with the Silver–Meal heuristic being one of the most well-known. It is used to determine lot sizes in contexts with variable demand; however, its performance deteriorates in the presence of periods with zero demand [30]. Other heuristic techniques include tabu search [31,32] and genetic algorithms [33], which have been applied to the lot-sizing problem through various solution space exploration strategies.

The issue of lot-sizing continues to be a relevant and actively researched topic. According to Hoseini et al. [34], the optimal lot-sizing policy in the supply chain plays an important role in industrial operations. Optimal lot sizes allow companies to reduce costs and deliver additional value to customers. In a recent study, Jeenanunta et al. [35] investigated inventory optimization and presented a simulation optimization technique to determine the optimal order level for a single-product inventory system. Wisniewski et al. [36] proposed a practical methodology for decision-making in inventory replenishment aimed at reducing holding costs. They presented a case study in which the results demonstrated the validity of the proposed method. Tobares et al. [37] developed a new optimization model to determine the optimal lot size, considering setup and holding costs as key variables. This analysis indicates that the order lot-sizing problem continues to be a subject of active scientific and technological interest.

Regarding the connection between physical systems and optimization, Kirkpatrick et al. [38] stated that there is a useful link between statistical mechanics, which studies the behavior of systems with multiple degrees of freedom in thermal equilibrium at a finite temperature, and multivariate or combinatorial optimization, understood as the search for the minimum of a function with respect to numerous parameters. Through a detailed analogy with the annealing process in solids, they demonstrated the possibility of building a robust conceptual framework to optimize the properties of large-scale and complex systems. This approach, known as Simulated Annealing (SA), provides a novel perspective for addressing traditional optimization problems.

In this context, a key concept is the Metropolis algorithm [39], which establishes a probabilistic rule, based on the Boltzmann distribution, for accepting or rejecting changes in the system’s configurations. This algorithm forms the foundation of simulated annealing, which, in addition to its probabilistic dynamics, requires the definition of a configuration (i.e., a set of possible solutions to the problem, often discrete and multidimensional) and a cost function, which allows for evaluating the relative performance of each proposed solution [40].

Recent studies have adapted and extended simulated annealing to specific problems in inventory planning and management. For instance, González-Ayala et al. [41] developed a modified simulated annealing (MSA) algorithm applied to a complex economic order quantity (EOQ) problem.

Similarly, Slama et al. [42] addressed the stochastic lot-sizing problem by proposing a sample average approximation approach based on Monte Carlo simulation for medium-sized instances and a hybrid Monte Carlo–genetic algorithm for large-scale cases.

With respect to previous research specifically addressing the lot-sizing problem using a lattice gas model and a cluster-based approach within the framework of statistical mechanics, no relevant studies were identified after applying a systematic search methodology. This methodology included the use of specific keywords, Boolean operators, and queries across recognized academic databases such as Scopus, Web of Science, and Google Scholar. The absence of previous studies in this area suggests a gap in the specialized literature, which reinforces both the originality and relevance of the present study.

On the other hand, entropy has garnered increasing interest across various scientific and management disciplines. In industrial engineering, the concept of entropy has been applied to cases such as decision tree analysis [43], workforce systems [44], logistics management [45], business process management [46], product life cycles [47], price–quality relationships [48], and the instantiation and execution of business processes [49].

In particular, within management science/operations research, some researchers have applied entropy-based approaches to account for disorder when modeling the behavior of production systems. Jaber et al. [50] proposed that the behavior of production systems closely resembles that of physical systems. This parallel suggests that improvements in production systems could be achieved by applying the first and second laws of thermodynamics to reduce system entropy (or disorder). To demonstrate the applicability of these laws, they used the economic order quantity (EOQ) model for production as an illustrative example.

Jaber [51] introduced an analogy between the behavior of production systems and physical systems by applying the concept of entropy cost to estimate some hidden or difficult-to-measure costs. Later, Jaber et al. [52] investigated the lot-sizing problem with learning and forgetting, incorporating the entropy cost concept into the work of Jaber and Bonney [53], who examined the effects of learning and forgetting in production lot-sizing problems with infinite and finite planning horizons.

Castellano [54] presented a study considering the continuous review reorder lot-sizing inventory model under stochastic demand. The study explored the performance of the maximum entropy principle to approximate the actual demand distribution during lead times. You et al. [55] investigated the lot-sizing problem with time-variable capacity in a rapidly changing production environment. The quality of the solution was evaluated in terms of entropy, and they proposed a preliminary method to calculate system entropy. They assumed that system entropy was the product of product structure complexity, the number of products produced, and the objective cost. However, they noted that future studies could develop a more reasonable and precise method for calculating system entropy.

These studies illustrate how concepts from different disciplines can be linked and combined in the analysis of complex systems.

The paper is organized as follows: The new approach linking a physical system with an optimization model is given in Section 3. In this section, the configurational mapping is introduced, establishing a transformation rule between the parameters involved in a one-dimensional lattice gas model with lateral interactions and those appearing in a self-developed model for determining the optimal order lot size. The basis of the cluster approximation is also provided in Section 3. The results, presented in Section 4, include classical quantities from adsorption theory, such as adsorption isotherms and configurational entropy of the adsorbed phase, now interpreted from the perspective of an order lot-sizing model. A new strategy for managing resources (monomers on adsorption sites or orders in planning periods) in response to environmental changes (chemical potential or unit order cost) is presented and discussed. Finally, the conclusions are drawn in Section 5.

## 3. Methodology

### 3.1. Lot-Sizing Model: Basic Definitions

Here, we study a single-item, *M*-period, order lot-sizing model. Let *N* denote the number of orders placed over the *M* periods, $C_{O}$ the fixed ordering cost per order, and $C_{H}$ the fixed holding cost per period for the quantity of material required for one period. The simplest strategy to meet the demand for the *M* periods consists of ordering the required quantity for each period at the beginning of that period (N=M). In this case, the order is consumed within the same period, resulting in no holding cost and a total cost of $C_{T}=MC_{O}$.

Introducing inventory storage (N<M) opens up various lot-sizing strategies. Identifying the optimal strategy that minimizes total cost is particularly valuable. Each strategy begins by ordering the material required for $n_{1}$ periods. After $n_{1}$ periods, these supplies are depleted, and additional material for $n_{2}$ periods is ordered. This process repeats iteratively until the *M* periods are covered. Therefore, each strategy can be represented by a set of *N* integers $\left\{n_{i}\right\}$ (i=1,…,N), satisfying

(1) $$\sum_{i=1}^{N}n_{i}=M.$$

**Figure 1:**
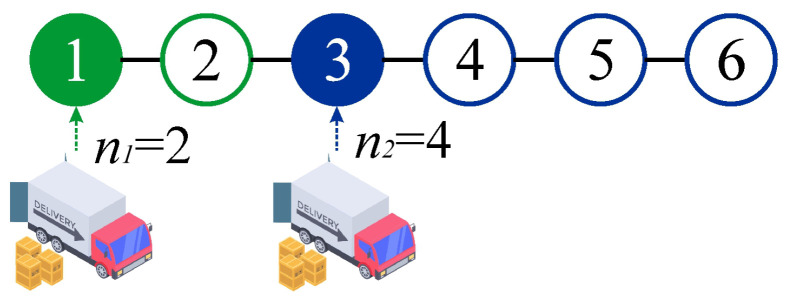
Requirement option with two orders N=2 of size $n_{1}=2$ and $n_{2}=4$ for a system with a planning horizon corresponding to the first half of the year, M=6.

As an example, Figure 1 presents an order planning system for the first half of the year (M=6), covering the period from January to June. In this case, two orders (N=2) are considered as part of the supply strategy. The first order is placed in January and covers the demand for that month and February ($n_{1}=2$). The second order is placed in March and fulfills the requirements for March, April, May, and June ($n_{2}=4$). The total cost associated with this strategy is given by the ordering and holding costs incurred throughout the six-month period, as expressed in the following equation:

(2) $$C_{T}\left(N=2,\left\{n_{1}=2,n_{2}=4\right\}\right)=2C_{O}+C_{H}+C_{H}+2C_{H}+3C_{H}.$$

On the right-hand side of Equation (2), the first term, $2C_{O}$, represents the total ordering cost for the two orders placed: one in January and another in March. The second term, $C_{H}$, corresponds to the holding cost of the material ordered in January, which remains in inventory during that month and is used in February. The third term, $C_{H}$, represents the holding cost of the material ordered in March, stored during that month and consumed in April. The fourth term, $2C_{H}$, reflects the holding cost of the material also ordered in March, kept in inventory during March and April, and used in May. Finally, the fifth term, $3C_{H}$, corresponds to the holding cost of the material ordered in March that remains in inventory throughout March, April, and May, and is consumed in June.

The application of this strategy is illustrated through a real case involving an industrial company engaged in the manufacturing of flexible plastic packaging, whose main raw material is white masterbatch. In this context, procurement is planned for the first half of the year, considering a six-month planning horizon (from January to June). The company incurs an ordering cost of USD 40.70 for each purchase order placed and a holding cost of USD 0.65 per unit stored per month.

(3) $$C_{T}\left(N=2,\left\{n_{1}=2,n_{2}=4\right\}\right)=2*40.70+0.65+0.65+2*0.65+3*0.65=85.95\$.$$

In general, for a strategy involving *N* orders and a set $\left\{n_{i}\right\}$, the total cost is expressed as

(4) $$C_{T}\left(N,\left\{n_{i}\right\}\right)=NC_{O}+C_{H}\sum_{i=1}^{N}\sum_{j=1}^{n_{i}-1}j.$$

### 3.2. Equivalence Between Lot-Sizing Model and Lattice Gas Model

In the framework of the lattice gas approximation, we assume that the surface is represented by a discrete lattice of *M* distinguishable sites. A lattice site represents an adsorptive potential minimum, where particles from a gas phase are allocated upon adsorption.

The substrate is exposed to an ideal gas phase at temperature *T* and chemical potential μ. Particles can be adsorbed on the substrate with the restriction of at most one adsorbed particle per site, and we consider a nearest-neighbor (NN) interaction energy *w* among them.

In order to describe a system of *N* particles adsorbed on *M* sites, we introduce the occupancy variable $c_{i}$, which can take the values $c_{i}=0$ if the site *i* is empty and $c_{i}=1$ if the site *i* is occupied by a particle. Under these considerations, the adsorbed phase is characterized by the Hamiltonian:

(5) $$H=N\left(\epsilon_{0}-\mu \right)+\frac{1}{2}w\sum_{i=1}^{M}\left(c_{i}\sum_{l\in \{NN,i\}}c_{l}\right)$$

where $\epsilon_{0}$ is the interaction energy between a particle and a lattice site, the factor of 1/2 is introduced to avoid double-counting, and “l∈{NN,i}” means that for a given site *i*, the sum runs over its NN sites.

As an example, consider a system of oxygen adsorbed in a 5A zeolite [56]: *N* denotes the number of O_2_ molecules adsorbed per 5A cavity; *M* is related to the volume of the cavity; *w* represents the O_2_–O_2_ intermolecular interaction energy in the adsorbed phase (in this case, w≈0.54 kcal/mol); $\epsilon_{0}$ is the interaction energy between the adsorbate and the substrate, with a value of $\epsilon_{0}=3.37$ kcal/mol for O_2_/5A; and μ is the chemical potential, which is related to the pressure controlling the experiment [57].

Let us now return to the lot-sizing problem and consider each operating period as a site in a lattice of size *M*, where a variable $c_{i}$ indicates whether an order is received in period (site) *i*. Specifically, $c_{i}=0$ if no order is received in period *i* and $c_{i}=1$ if an order is received. We also introduce the variable $d_{i}$, associated with site *i*, defined as follows: if $c_{i}=0$, then $d_{i}=0$; if $c_{i}=1$, then $d_{i}$ represents the number of periods in which no orders were received between order *i* and the next period with an order, or, if order *i* is the last order placed, between order *i* and the end of the planning horizon. Using these newly introduced variables, the total cost function in Equation (4) can be rewritten as follows:

(6) $$C_{T}=NC_{O}+C_{H}\sum_{i=1}^{M}\left(c_{i}\sum_{j=1}^{d_{i}}j\right).$$

As an example, let us revisit the case shown in Figure 1 using the variables $c_{i}$ and $d_{i}$. It is straightforward to deduce that $c_{1}=c_{3}=1$, $c_{2}=c_{4}=c_{5}=c_{6}=0$, $d_{1}=1$, $d_{3}=3$, and $d_{2}=d_{4}=d_{5}=d_{6}=0$. Thus, applying Equation (6), we obtain

(7) $$\begin{array}{ccc} C_{T} & = & 2C_{O}+C_{H}\left[\left(c_{1}\sum_{j=1}^{d_{1}=1}j\right)+\left(c_{3}\sum_{j=1}^{d_{3}=3}j\right)\right]\\ & = & 2C_{O}+C_{H}+C_{H}+2C_{H}+3C_{H}, \end{array}$$

consistent with the previously derived result in Equation (2).

By comparing the Hamiltonian in Equation (5) with the total cost function in Equation (6), it becomes clear that an isomorphism exists between the lattice gas model and the lot-sizing model. Specifically, the first term of the total cost function, which accounts for the ordering costs, can be identified with the adsorbate–substrate adsorption energy in the lattice gas model. On the other hand, the second term of the total cost function, which represents holding costs, corresponds to the lateral adsorbate–adsorbate interaction energy in the lattice gas model. The complete mapping between the lattice gas and lot-sizing models is presented in Table 1.

The lattice gas model, along with its equivalent Ising model for magnetism, has been extensively studied in the literature, with its results validated across a wide range of systems [57,58,59]. Our goal was to incorporate the lot-sizing model into this well-established framework, leveraging its solid and validated library of results. This not only enhances the theoretical foundation of the model presented here but also provides a framework for generalizing studies to multi-item lot-sizing problems, cases of variable material requirement per period, and other related extensions.

### 3.3. Cluster Approximation

As we mentioned at the end of the previous section, there are proven theories for studying the Ising model and the lattice gas model [57,58,59]. One of them is called the cluster approximation (CA) [16,17,18,19,20,21], and it is the approximation that will be used here.

The fundamental assumption on which CA is based is that the system can be considered as a repetition of identical small subsystems or clusters. Then, the solution of the system in the thermodynamic limit is constructed from the exact solution of a small lattice (cluster). The application of this approximation can be visualized as follows: An image of the initial lattice is constructed through its partition into a set of clusters, where each cluster is a subset of *m* sites. Then, with an adequate choice of the size and shape of the cluster, the log of the grand partition function per spin of the complete system can be well approximated by the log of the grand partition function of the cluster.

**Figure 2:**
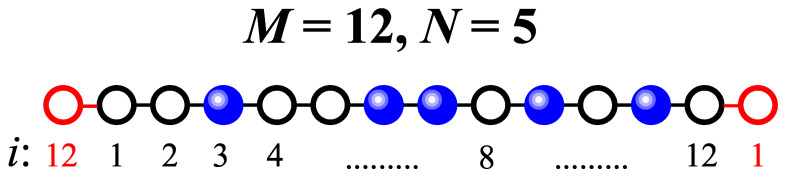
Schematic representation of a small cell of size M=12 sites with N=5 adsorbed particles.

Building on the reasoning introduced in the previous paragraph, and considering a lattice gas model of interacting particles, we assume that deposition occurs on a small, one-dimensional lattice (the cluster) consisting of *M* sites. Additionally, periodic boundary conditions are applied. As an example, Figure 2 illustrates a cluster with M=12 adsorption sites (empty black circles) and N=5 adsorbed particles (solid blue spheres). Periodic boundary conditions are implemented by adding an extra neighboring site at each end of the lattice, as indicated by the empty red circles. Similarly, solid black lines correspond to interactions in the cluster and solid red lines represent boundary couplings.

The grand partition function of a cluster of size *M* is given by

(8) $$\Xi =1+\sum_{N=1}^{M}\lambda^{N}\sum_{E}g\left(E,N\right)\exp\left(-E/k_{B}T\right),$$

where $\lambda \equiv \exp\left(\mu /k_{B}T\right)$ is the activity of the adsorbed species, $k_{B}$ is the Boltzmann constant, and $g\left(E,N\right)$ is the number of configurations corresponding to the *N* adsorbed particles with the same energy *E* (*E* depends on *N* and the number of interaction pairs on the cluster). $g\left(E,N\right)$ can be calculated exactly using a computer algorithm.

The adsorption isotherm and the configurational entropy per site of the adsorbed phase *s* can be calculated from Equation (8) [57],

(9) $$\theta \left(\mu \right)=\frac{k_{B}T}{M}{\left(\frac{\partial \ln\Xi }{\partial \mu }\right)}_{T},$$

and

(10) $$\frac{s}{k_{B}}=\frac{T}{M}{\left[\frac{\partial \ln\Xi (M,\lambda ,T)}{\partial T}\right]}_{M,\lambda }+\frac{1}{M}\ln\Xi \left(M,\lambda ,T\right),$$

where θ(μ), commonly referred to as the adsorption isotherm in the framework of the lattice gas model, represents the surface coverage as a function of the chemical potential. Using the mapping rules in Table 1, the quantities defined in Equations (9) and (10) can be reinterpreted in terms of the lot-sizing model. This point will be discussed in detail in the next section.

Before concluding this section, we would like to highlight three points. First, following historical precedent, we will use θ(γ) instead of θ(μ) when referring to the lot-sizing problem, where $\gamma =2C_{O}/C_{H}$ is a characteristic parameter commonly used in lot-sizing theory [25].

Second, and as mentioned above, in a lattice gas with *M* distinguishable sites, between 1 and *M* monomers can be adsorbed. Similarly, in the supply model with a planning horizon *M*, between 1 and *M* orders can be placed. To count the possible configurations of the system, the following considerations must be taken into account.

The requirement to have raw materials available from the first execution period imposes a condition on the system’s possible configurations. In the language of the lattice gas model, this condition implies that the first site of the system must be occupied. Thus, in the case of a single order (N=1), where the order size includes the material required for all periods in the system, there will be only one possible configuration for this case: the first site is occupied and the rest of the sites are empty. In the general case of *N* orders (with 2≤N≤M), the count of possible configurations arises by placing the first order at the first site of the lattice (first site occupied), while the remaining N−1 orders can be distributed among the remaining M−1 periods or available sites. The total number of possible configurations is then given by M−1N−1=(M−1)!/(N−1)!(M−N)!.

Third, we emphasize that, in the lot-sizing problem, the system is inherently discrete, and the planning horizon typically ranges from M≈7 to M≈30. This characteristic makes the cluster approximation particularly well-suited for this problem, leading to exact results.

### 3.4. Summary of the Modeling Process

The modeling procedure described in Section 3 can be summarized in the following steps:

(1) Select the specific lot-sizing problem to be studied.
(2) Based on the selected problem in step (1), choose the corresponding equivalent lattice gas model. An example is shown in Table 1 for the single-item lot-sizing model considered in this work. Other problems, such as multi-item lot-sizing or cases involving variable material requirements per period, will require a different definition of lateral interactions and a modified number of adsorbed components.
(3) Apply cluster approximation. To do this, the factor g(E,N) must be obtained using a computational algorithm that explores all possible configurations. In our case, a C++ code developed by the authors was used to count all possible arrangements of *N* orders over a planning horizon of length *M*. Once g(E,N) is determined, the grand partition function is computed, from which all relevant statistical properties can be derived.

## 4. Results

Cluster approximation was applied to the lot-sizing model to analyze its behavior from a statistical mechanical perspective.

In the standard lattice gas model, one of the most extensively studied quantities is the adsorption isotherm θ(μ), as given in Equation (9) [57]. This quantity is not only of theoretical interest but also represents the most frequently measured property in experimental studies of surface physics. For this reason, we begin by analyzing the equivalent of the adsorption isotherm in the context of the lot-sizing problem.

**Figure 3:**
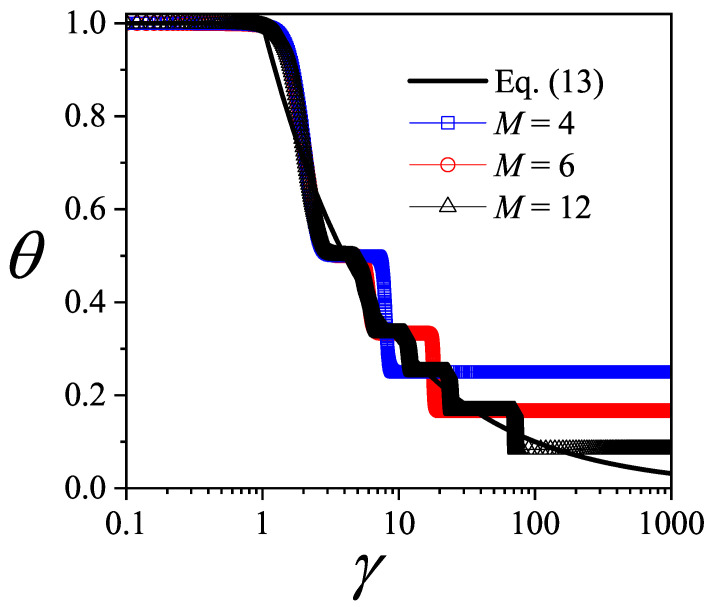
θ=N/M as a function of γ (log scale) for three different values of *M* as indicated in the inset.

In Figure 3, θ=N/M is plotted as a function of γ (in log scale) for three different values of *M*: 4 (blue squares); 6 (red circles); and 12 (black triangles). Symbols joined by lines represent analytical CA results. In our calculations, we set $C_{H}=10$ (in arb. units) and varied $C_{O}$.

The overall behavior of the curves in Figure 3 can be explained as follows: At very low values of γ, the ordering cost was negligible compared to the holding cost, which led to the maximum number of orders (N=M) and, accordingly, θ=1. This trend persisted until approximately γ≈1, where the ordering cost became significant, causing the number of orders to decrease. In the opposite limit, when γ was very large, the ordering cost was significantly higher than the holding cost. As a result, the number of orders reached its minimum (N=1), leading to θ=1/M for γ→∞. As observed in the different curves, the intermediate regime of γ values was characterized by the presence of plateaus in the function θ(γ). We refer to these flat regions below.

The plateaus observed in Figure 3 have precedents in classical problems of surface physical chemistry [60,61]. Specifically, in the case of a one-dimensional lattice gas model with repulsively interacting adsorbates, the resulting adsorption isotherms are characterized by the presence of plateaus corresponding to the formation of distinct adsorbate structures [60,61]. These structures depend on the shape of the adsorbate and the characteristics of lateral interactions, such as their range, additivity, and other factors.

**Figure 4:**
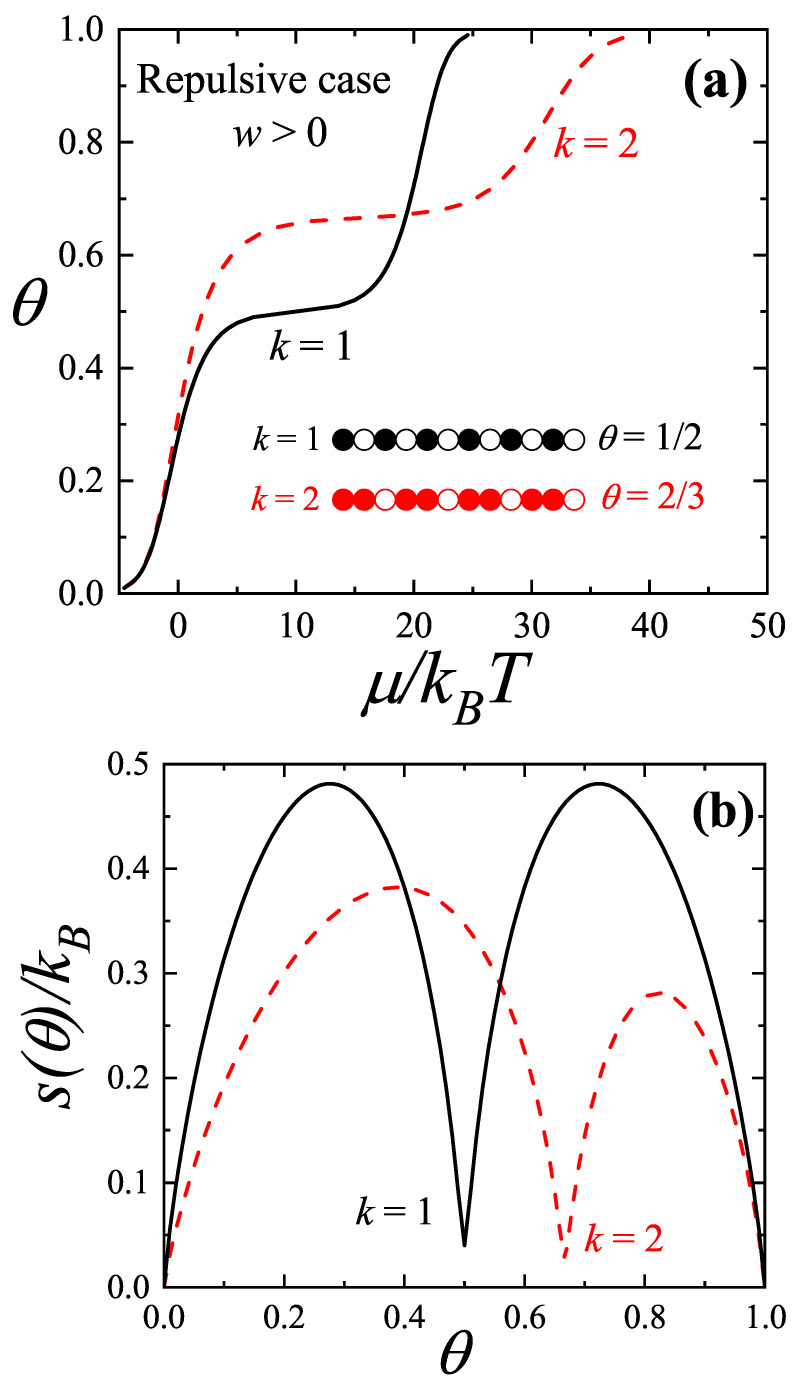
(**a**) Lattice coverage θ as a function of the reduced chemical potential $\mu /k_{B}T$ for repulsive monomers (k=1, solid black line) and dimers (k=2, dashed red line), with interaction strength $w/k_{B}T=10$. The inset illustrates the ordered surface structures that emerged for k=1, θ=1/2 and k=2, θ=2/3. (**b**) Entropy per site, *s* (in $k_{B}$ units), versus lattice coverage, θ, for the same cases displayed in part (**a**).

As an illustrative example, Figure 4a depicts the adsorption isotherms of repulsive monomers and dimers in a one-dimensional system in a low temperature regime, highlighting the different phases that emerge. The problem of *N* adsorbed *k*-mers on a one-dimensional lattice with *M* sites, including a nearest-neighbor interaction energy *w* between the ends of adjacent *k*-mers, is exactly solved [60,62,63]. As shown in Appendix A, the chemical potential (μ) and the entropy per site (*s*) can be explicitly determined as functions of coverage.

The curves in Figure 4 were obtained from Equations (A9) and (A10) with $w/k_{B}T=10$. In the case of the adsorption isotherm for monomers (k=1, solid black line in Figure 4a), particles avoiding configurations with repulsive nearest neighbors were ordered in a structure of alternating adsorbed atoms separated by an empty site at θ=1/2. The width of the step was directly proportional to the energy per particle necessary to alter such an ordered structure. As the size of adsorbed particles increased, the step shifted to higher coverages. Based on analogous arguments to the ones given for monomers, it was straightforward that the step was located at θ=2/3 for dimers (k=2, dashed red line). The inset in Figure 4a illustrates the ordered surface structures that emerged for k=1, θ=1/2 and k=2, θ=2/3. Open circles indicate vacant sites, filled black circles represent adsorbed monomers, and filled red circles denote adsorbed dimers.

On the other hand, the configurational entropy curves of the adsorbed phase (see Figure 4b) exhibited local minima at θ=1/2 for monomers and θ=2/3 for dimers. These entropy minima originated from the specific structural arrangements associated with the adsorption isotherms, as discussed above and illustrated in the inset of Figure 4a.

**Figure 5:**
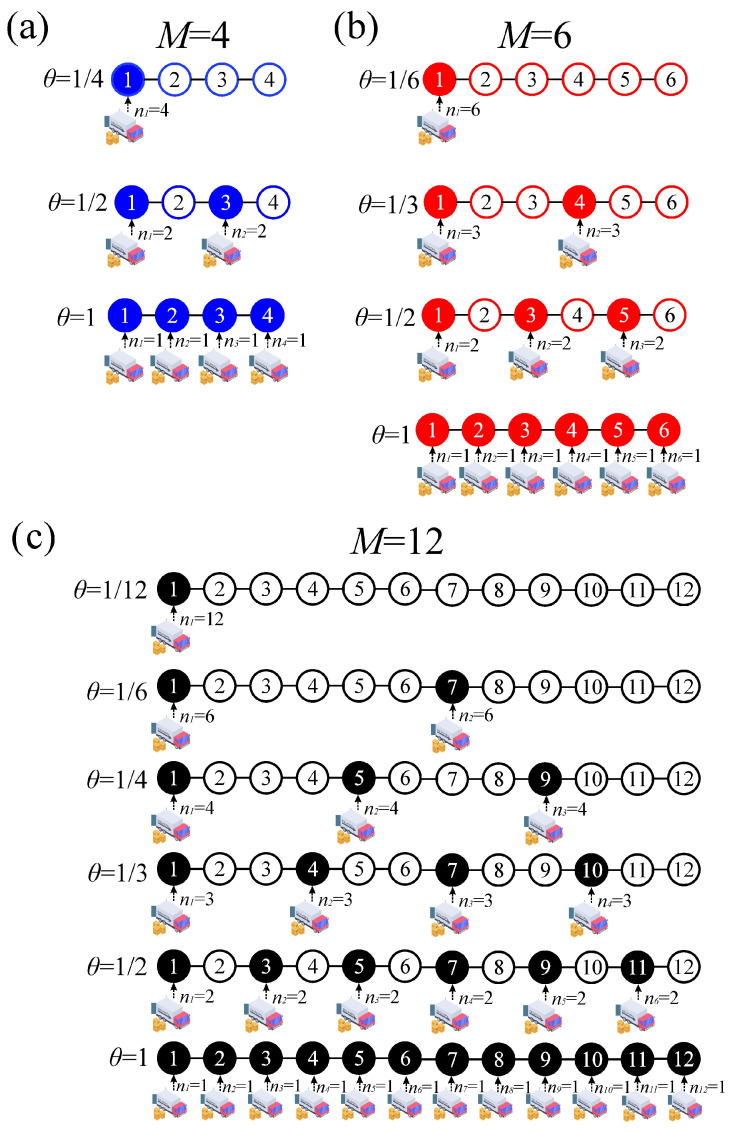
Snapshots illustrating the ordered configurations observed for M=4 (panel (**a**)), M=6 (panel (**b**)), and M=12 (panel (**c**)).

Let us now return to the curves shown in Figure 3 and examine the plateaus that appeared, along with their connection to the formation of ordered structures in the equivalent lattice gas model. We begin with the case of M=4. In addition to the trivial cases observed at θ=1/4 (N=1) and θ=1 (N=4), a distinct plateau was observed at intermediate coverage, specifically at θ=1/2 (N=2). This plateau corresponded to the ordered configuration illustrated in Figure 5a. For M=4, no other value of *N* yielded a configuration with evenly spaced adsorbates. In the case of M=6, intermediate plateaus emerged at N=3 (θ=1/2) and N=2 (θ=1/3), as shown in Figure 5b. Finally, for M=12, plateaus were observed at N=6 (θ=1/2), N=4 (θ=1/3), N=3 (θ=1/4), and N=2 (θ=1/6). Representative snapshots of the corresponding ordered structures are presented in Figure 5c.

In a recent study by our group [25], an optimization model for the lot-sizing problem was introduced based on a physical system approach. By establishing that the material supply problem was isomorphic to a one-dimensional mechanical system of point particles connected by elastic elements, an exact solution was obtained, and the cost optimization conditions emerged naturally. The results indicate that for a given *N*, the optimal strategy to minimize the total cost involves placing the *N* orders at equal intervals. In the context of the lattice gas model, this prediction aligns with the structures derived from the isotherms in Figure 3.

To further support the arguments presented in the previous paragraph, we computed the adsorption energy of the adsorbed layer in the lattice gas model for each coverage where plateaus formed. These energies were equivalent to the total cost in the terminology of the lot-sizing model. For simplicity, we restricted our analysis to the energy component that accounted only for lateral interactions (holding costs in the lot-sizing model). The total cost and holding cost differed only by an additive term equal to $NC_{O}$. The results are reported in Table 2. The values in the table, obtained using CA from the equivalent lattice gas model, matched exactly with the exact values derived from the following equation:

(11) $$C_{H,0}\left(\theta \right)=\frac{C_{H}}{2}\left(\frac{1}{\theta }-1\right),$$

where $C_{H,0}\left(\theta \right)$ represents the minimum holding cost for a given θ (*N*). Equation (11) was obtained from Ref. [25] considering equally spaced orders.

In Ref. [25], the optimal number of orders, $N_{\mathrm{op}}$, for which the total cost reached a minimum was exactly calculated as a function of γ:

(12) $$N_{\mathrm{op}}\left(\gamma \right)=\left\{\begin{array}{ccc} M & \mathrm{if} & \gamma \leq 1\\ \gamma^{-1/2}M & \mathrm{if} & \gamma >1\;\;. \end{array}\right.$$

By normalizing with the planning horizon *M*, we obtained

(13) $$\theta_{\mathrm{op}}\left(\gamma \right)=\frac{N_{\mathrm{op}}\left(\gamma \right)}{M}=\left\{\begin{array}{ccc} 1 & \mathrm{if} & \gamma \leq 1\\ \gamma^{-1/2} & \mathrm{if} & \gamma >1\;\;. \end{array}\right.$$

The function $\theta_{\mathrm{op}}\left(\gamma \right)$ (Equation (13)) is shown in Figure 3 as a black solid line. As can be observed, the curves obtained for discrete values of *M* converged to the exact solution given by Equation (13) in the limit of M→∞. This result indicates that the curves presented in Figure 3 provide the optimal value of *N* for each γ, thus completing the interpretation of the function θ(γ) derived from the thermodynamic analogy with the lattice gas model.

**Figure 6:**
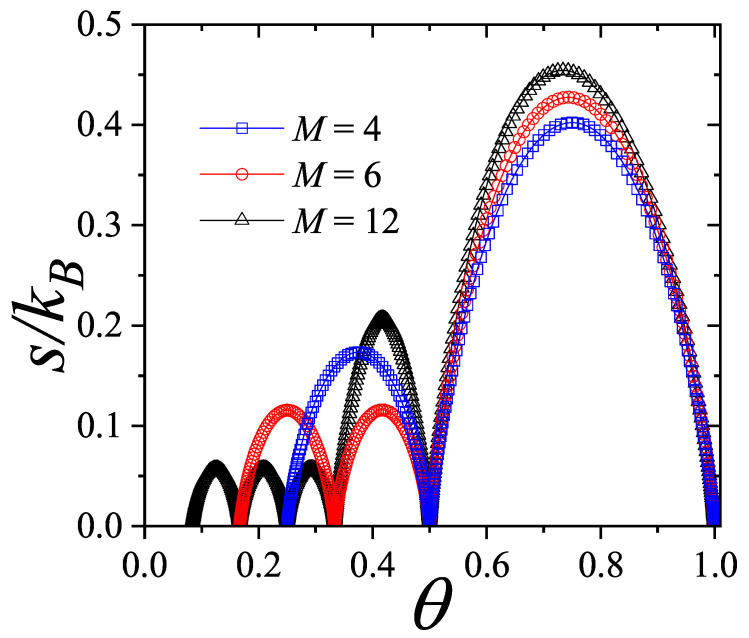
Entropy per site *s* (in $k_{B}$ units) as a function of θ for the same cases analyzed in Figure 3. Here, the curves were derived using Equation (10).

The structures discussed in terms of the function θ(γ) could also be analyzed from an entropy perspective. To this end, using CA theory, we computed the entropy as a function of coverage (Equation (10)) for the same cases reported in Figure 3. The results are shown in Figure 6, with the same symbology as in Figure 3.

Unlike the entropy curves derived from the lattice gas model (Figure 4b), which started at θ=0 and $s/k_{B}=0$ (as only one configuration existed for an empty lattice), the curves shown in Figure 6 began at θ=1/M due to the constraint discussed in Section 3.3. Specifically, the requirement that raw materials be available from the first planning period implied that the case N=0 was not allowed in the lot-sizing model. Moreover, for N=1, the only feasible configuration was one in which the first site of the system was occupied, meaning that the entire demand across the planning horizon was satisfied in the first period. Since only one configuration existed for N=1, the entropy also started from zero at θ=1/M, i.e., $s/k_{B}=0$. This initial occupancy condition also broke the particle–vacancy symmetry present in the lattice gas model. As a result, quantities such as θ(γ) (Figure 3) and s(θ) (Figure 6) became asymmetric with respect to θ=1/2.

Depending on the value of *M*, additional zeros appeared in the entropy curves, as shown in Figure 6. The pronounced minima observed in these curves aligned with the formation of the ordered structures previously discussed and illustrated in Figure 5. To interpret these minima, it is helpful to revisit the lattice gas model. The configurations shown in the inset of Figure 4a correspond to arrangements in which no nearest-neighbor interactions occurred between different adsorbed particles. In other words, due to the repulsive nature of the coupling constant *w*, the configurations responsible for the entropy minima in a lattice gas were those that completely eliminated lateral interaction energy, zero in the example of Figure 4. Any alternative configuration at the same coverage would have had a higher configurational energy. Analogously, in the lot-sizing model, the configurations shown in Figure 5 correspond to those that minimized the total cost of the procurement strategy for a given *N* and specific purchasing and storage parameters. When θ varied between two entropy minima, the number of feasible configurations first increased as more supply strategies became accessible, reached a maximum, and then, due to symmetry, decreased again to zero at the next minimum.

The results derived from the analysis of Figure 4, Figure 5 and Figure 6 corroborate the arguments presented in Ref. [25]. These findings further support the conclusion that for a fixed *N*, the most cost-effective strategy consists of distributing the *N* orders at equal intervals. In other words, the structures emerging from the plateaus of the isotherms, or equivalently, from the minima of the entropy per site (see Figure 5), arose from the minimization criteria imposed by thermodynamics and corresponded to the optimal purchasing and storage strategies in the associated lot-sizing model [25].

Our study builds upon existing research that applies concepts from statistical physics to optimization problems, particularly the use of lattice gas models in supply chain management. While previous works, such as those by Jaber et al. [50,51,52,53] and Castellano [54], introduced entropy-based approaches and thermodynamic analogies to production systems, this is the first comprehensive application of a lattice gas framework specifically to the classical order lot-sizing problem. By establishing a formal analogy with well-understood physical systems, our approach advances the theoretical foundation of supply chain modeling, offering novel analytical tools such as free energy minimization and configurational entropy as decision metrics. This interdisciplinary integration not only expands the methodological repertoire but also provides deeper insights into the structural properties of optimal policies beyond traditional heuristics or deterministic models.

The proposed framework has significant practical implications for supply chain managers and decision-makers seeking robust and flexible ordering policies. By quantifying system complexity and robustness through configurational entropy, our approach enables practitioners to evaluate the stability of different inventory strategies. Companies can benefit from this methodology to improve risk management and adaptivity, while operational researchers might employ the model to identify equilibrium-like states that minimize costs and variability. Ultimately, the insights derived from this thermodynamic perspective could inform the development of decision support systems that incorporate probabilistic and physical analogies for enhanced strategic planning.

The results presented in this section reproduce previous exact calculations reported in Ref. [25], demonstrating that mapping the lot-sizing problem onto a lattice gas model, combined with well-established cluster approximation, provides a robust and coherent framework for addressing supply chain challenges. More importantly, this approach introduces a novel perspective: reinterpreting purchasing and storage strategies as thermodynamic states of a physical system. This innovative viewpoint leverages fundamental physical concepts such as energy and entropy, concepts that have not been previously considered in classical inventory models. By framing the problem in this way, we open new avenues for research, potentially enabling the extension of this framework to more complex and realistic scenarios. This promising direction will be further explored in the following section.

Despite its promising theoretical and practical contributions, our study has certain limitations that should be acknowledged. The current model primarily addresses single-item, deterministic demand scenarios, and the extension to multi-item or stochastic environments remains an open challenge. Additionally, while the cluster approximation provides accurate estimations, it may become computationally intensive for very large-scale problems or more complex interaction structures. Future research should aim to relax some of the simplifying assumptions, develop efficient algorithms for rapid configuration computation, explore the integration of dynamic elements such as learning effects and supply disruptions, and validate the model using real-world case studies. These efforts will be crucial to fully realize the potential of statistical physics-based modeling in operational decision-making and address the identified limitations.

## 5. Conclusions

In this work, we introduced a novel framework for analyzing the classical lot-sizing problem by mapping it onto a lattice gas model. This approach allowed us to leverage the formalism of statistical mechanics, traditionally used in the study of thermodynamic systems, in the context of supply chain optimization.

At the core of our methodology lies the characterization of each period within the planning horizon through a discrete variable $c_{i}$, where $c_{i}=0$ denotes no order and $c_{i}=1$ indicates that an order is placed in period *i*. Based on this representation, we showed that it is possible to construct a Hamiltonian for the lot-sizing problem and establish an isomorphism with well-known models in statistical mechanics, such as lattice gas models of adsorbed particles and the Ising model of lattice spins. This study focused primarily on the lattice gas formulation, leveraging our group’s extensive experience in modeling adsorption phenomena.

This interdisciplinary perspective enables the reinterpretation of purchasing strategies as thermodynamic states, from which quantities such as energy and entropy, previously absent from classical supply chain models, can be derived. This constitutes a significant theoretical contribution. Additional key results of this work include the following:

- Optimal strategies emerge from thermodynamic principles: We demonstrated that optimal supply policies naturally arise from the minimization of a free energy function, mirroring the principle used to determine equilibrium states in physical systems. This insight opens a promising research avenue at the intersection of statistical physics and supply chain optimization.
- Accurate cost prediction through cluster approximation: Using cluster approximation techniques, we confirmed that crucial metrics, such as total cost and order distributions, can be predicted with high accuracy, aligning closely with known exact solutions [25].
- Configurational entropy as a complexity metric: Our analysis highlights the role of configurational entropy as a measure of complexity and robustness in inventory strategies, introducing a novel and meaningful decision-making metric. Specifically, for the physical model described in Ref. [25], which consists of a one-dimensional mechanical system of point particles connected by elastic elements, the optimal strategy to minimize the total cost for a given *N* corresponds to a configuration in which all springs have equal length (constant $n_{i}$), resulting in zero net force on each particle. Within the framework of the lattice gas model, this exact result is consistent with the structures inferred from the curves of configurational entropy as a function of coverage.
- A powerful analytical tool for supply planning: As a corollary of the above results, we showed that the theoretical machinery developed for studying adsorption and magnetism via lattice gas models offers a simple yet powerful tool for identifying optimal strategies in supply planning. This is a particularly valuable contribution, as it introduces to supply chain management a rich set of analytical tools from statistical physics. As a concrete example, we demonstrated that cluster approximation theory not only reveals the structure of optimal strategies but is especially effective due to the discrete nature of the planning horizon *M*.

Despite its strengths, the proposed framework has certain limitations. These include the simplified modeling of interactions and challenges in scaling the approach to settings with long planning horizons (i.e., large values of *M*) and multi-item lot-sizing scenarios. In such cases, practical implementation would require the development of efficient algorithms capable of computing the number of configurations *g* (Equation (8)) within reasonable time frames. Work in this direction is currently underway.

Future work will focus on extending this theoretical framework to more complex settings, including multi-item lot-sizing problems and scenarios with time-dependent demand. In the former case, the problem will be mapped onto a multicomponent lattice gas model, with each species representing a distinct product. In the latter, we will consider lattice gas models with non-additive interactions to account for the temporal variability in material requirements. These developments have the potential to influence both scholarly understanding and practical applications in inventory control and logistics.

## Figures and Tables

**Table 1:** Rules for the mapping from a lot-sizing model to a lattice gas model.

Lot-Sizing Model	Lattice Gas Model
*M*, planing horizon	*M*, lattice size
*N*, number of orders	*N*, number of adsorbed particles
$C_{O}$, ordering cost	$\left(\epsilon_{0}-\mu \right)$, effective chemical potential, adsorbate–adsorbent interaction
$C_{H}$, holding cost	adsorbate–adsorbate interaction

**Table 2:** Values of the holding cost (lateral adsorbate–adsorbate interaction energies in the language of lattice gas models) for each coverage where plateaus formed in the systems studied in Figure 3. The numbers in parentheses indicate the coverage corresponding to each value of *M* and *N*.

*N*	*M* = 4	*M* = 6	*M* = 12
1	15 (0.25)	25 ($\overset{◠}{0.16}$)	55 ($\overset{◠}{0.083}$)
2	5 (0.50)	10 ($\overset{◠}{0.3}$)	25 ($\overset{◠}{0.16}$)
3	…	5 (0.50)	15 (0.25)
4	…	…	10 ($\overset{◠}{0.3}$)
6	…	…	5 (0.50)

## Data Availability

Dataset available upon request to the authors.

## Appendix A. One-Dimensional Lattice Gas of Interacting *k*-mers

In this appendix, we examine the adsorption behavior of linear *k*-mers on one-dimensional homogeneous lattices. Each *k*-mer consists of *k* identical units, each occupying one site upon adsorption. The interaction energy between adjacent *k*-mer units is denoted as *w*, applicable when their ends are nearest neighbors. The lattice constant is represented by *a*, ensuring that exactly *k* sites are occupied by a *k*-mer. For simplicity, the interaction energy between a *k*-mer unit and a lattice site is considered negligible.

Let *N* linear *k*-mers be adsorbed on *M* lattice sites, with nearest-neighbor interaction energy *w* between *k*-mer ends. The coverage is given by θ=kN/M. We introduce a mapping from the original lattice L to an effective lattice $L^{\prime }$, where each empty site in L corresponds to an empty site in $L^{\prime }$, and each group of *k* occupied sites by a *k*-mer in L is represented by a single occupied site in $L^{\prime }$. Consequently, the total number of sites in $L^{\prime }$ is

(A1) $$M^{\prime }=M+\left(k-1\right)N,$$

and the coverage of $L^{\prime }$ is

(A2) $$\theta^{\prime }=N/M^{\prime }=\left(\theta /k\right)/\left[1-\frac{\left(k-1\right)}{k}\theta \right].$$

The canonical partition functions Q(kN,M,T), $Q^{\prime }\left(N,M^{\prime },T\right)$ in the original and effective lattice must be equal. Thus,

(A3) $$Q\left(kN,M,T\right)=\sum_{\left\{X\right\}}\exp\left[-\frac{E(X)}{k_{B}T}\right]=Q^{\prime }\left(N,M^{\prime },T\right)=\sum_{\left\{X^{\prime }\right\}}\exp\left[-\frac{E(X^{\prime })}{k_{B}T}\right],$$

where {X} and $\left\{X^{\prime }\right\}$ refer to a sum over all possible configurations in L and $L^{\prime }$, respectively.

Accordingly, the Helmholtz free energies per site in L and $L^{\prime }$ and *f* and $f^{\prime }$, respectively, are related through

(A4) $$\begin{array}{ccc} f(kN,M,T) & = & -\frac{k_{B}T}{M}\ln\left[Q\left(kN,M,T\right)\right]\\ & = & -\frac{k_{B}T}{M}\ln\left[Q^{\prime }\left(N,M^{\prime },T\right)\right]\\ & = & \frac{M^{\prime }}{M}f^{\prime }\left(N,M^{\prime },T\right)\\ f(kN,M,T) & = & \frac{M^{\prime }}{M}f^{\prime }=\left\{1+\left[\frac{\left(k-1\right)}{k}\theta \right]\right\}f^{\prime }\left(N,M^{\prime },T\right). \end{array}$$

This relationship makes complete the mapping from the original problem of *k*-mer adsorption on L to an effective Ising-like one (monomer adsorption) on $L^{\prime }$. $f^{\prime }$ can then be written in terms of the probability *y* of having two nearest-neighbor sites occupied in $L^{\prime }$, by means of the cumulant variation method [62,63], as

(A5) $$\begin{array}{ccc} f\left(\frac{N}{M^{\prime }},T\right) & = & wy-k_{B}T\left[\theta^{\prime }\ln\theta^{\prime }+\left(1-\theta^{\prime }\right)\ln\left(1-\theta^{\prime }\right)-y\ln y\right]\\ & - & k_{B}T\left[-2\left(\theta^{\prime }-y\right)\ln\left(\theta^{\prime }-y\right)-\left(1-2\theta^{\prime }-y\right)\ln\left(1-2\theta^{\prime }-y\right)\right]. \end{array}$$

For each set of values $\theta^{\prime },T$, *y* is obtained by minimizing $f^{\prime }$. Thus,

(A6) $$y=\theta^{\prime }-\left(\frac{A}{2}\right)+{\left[\frac{A^{2}}{4}-\theta^{\prime }\left(1-\theta^{\prime }\right)A\right]}^{1/2},$$

where $A={\left[1-\exp\left(-w/k_{B}T\right)\right]}^{-1}$. In the infinite temperature limit $w/k_{B}T\to 0$, $A\approx k_{B}T/w$ and $y\approx {\theta^{\prime }}^{2}+O\left(w/k_{B}T\right)$, as expected for a totally random distribution of units on the lattice. For infinitely repulsive interactions $w/k_{B}T\to \infty$, A→1 and y=0 if $\theta^{\prime }\leq 1/2$ (i.e, no two nearest-neighbor occupied sites are present on the lattice) or $y=2\theta^{\prime }-1$ if $\theta^{\prime }\geq 1/2$. For infinitely attractive interactions, $w/k_{B}T\to -\infty$, this yields $y=\theta^{\prime }$, as physically expected.

By using the relationship between $\theta^{\prime }$ and θ [from Equation (A2)] in Equation (A6) and replacing Equation (A6) in Equation (A5), the exact form of *f* is obtained:

(A7) $$\begin{array}{ccc} f(\theta ,T) & = & w\left[\frac{\theta }{k}-\alpha \right]-k_{B}T\left[\frac{\theta }{k}\ln\frac{\theta }{k}+\left(1-\theta \right)\ln\left(1-\theta \right)-2\alpha \ln\alpha \right]\\ & - & k_{B}T\left\{-\left[\frac{\theta }{k}-\alpha \right]\ln\left[\frac{\theta }{k}-\alpha \right]-\left(1-\theta -\alpha \right)\ln\left(1-\theta -\alpha \right)\right\}, \end{array}$$

where α is given by

(A8) $$\alpha =\frac{2\theta (1-\theta )}{k\left[1-\frac{\left(k-1\right)}{k}\theta +b\right]}\;\;\;\;\;\;\mathrm{and}\;\;\;\;\;\;b={\left\{{\left[1-\frac{\left(k-1\right)}{k}\theta \right]}^{2}-\frac{4}{kA}\left(\theta -\theta^{2}\right)\right\}}^{1/2}.$$

The coverage dependence of the chemical potential and the entropy per site *s* can be straightforwardly obtained from derivatives of the free energy [57]:

(A9) $$\begin{array}{ccc} \mu /k_{B}T=k{\left(\frac{\partial f}{\partial \theta }\right)}_{M,T} & = & w/k_{B}T+\ln\left[k(b-1+\theta )+\theta \right]+\left(k-1\right)\ln\left[1-\frac{\left(k-1\right)}{k}\theta +b\right]\\ & + & \left(k-1\right)\ln k-\ln\left[k(b+1-\theta )-\theta \right], \end{array}$$

and

(A10) $$\begin{array}{ccc} s/k_{B}=-{\left(\frac{\partial f}{\partial T}\right)}_{M,N} & = & \frac{\theta }{k}\ln\frac{\theta }{k}+\left(1-\theta \right)\ln\left(1-\theta \right)-2\alpha \ln\alpha \\ & - & \left[\frac{\theta }{k}-\alpha \right]\ln\left[\frac{\theta }{k}-\alpha \right]-\left(1-\theta -\alpha \right)\ln\left(1-\theta -\alpha \right). \end{array}$$
